# Myxofibrosarcoma of the Breast: A Case Report

**DOI:** 10.7759/cureus.39046

**Published:** 2023-05-15

**Authors:** Keigo Amaya, Akira Okimura, Hiroshi Hirano, Midori Wakiya, Yumika Ito, Kimito Yamada, Munehide Nakatsugawa

**Affiliations:** 1 Breast Surgery, Tokyo Medical University Hachioji Medical Center, Hachioji, JPN; 2 Diagnostic Pathology, Tokyo Medical University Hachioji Medical Center, Hachioji, JPN

**Keywords:** a case report, sarcoma of breast, breast non-epithelial tumors, myxofibrosarcoma, malignant tumors

## Abstract

Development of a myxofibrosarcoma in the breast tissue is extremely rare. Reported here is a case of myxofibrosarcoma found in the left breast tissue of a male in his late fifties. The patient first underwent tumor resection, followed by a left mastectomy with the reconstruction of the vastus lateralis valve. The tumor comprised atypical spindle-shaped cells in a myxoid matrix with elongated blood vessels. Myxofibrosarcoma was diagnosed based on histology and immunohistochemical examination results performed for differential diagnosis. At two years and two months after the mastectomy, no local occurrence or metastasis had occurred.

## Introduction

Myxofibrosarcoma generally develops in the extremities, though it sometimes occurs in the head or neck. On the other hand, a myxofibrosarcoma in the breast tissue is extremely rare, with only 18 known cases reported thus [1-9]. Since there are no specific markers for myxofibrosarcoma, diagnosis of occurrence in the breast tissue has been based on histology in most cases. We report a case of myxofibrosarcoma in the breast tissue with immunohistochemical examination results for differential diagnosis.

## Case presentation

**Figure 1:**
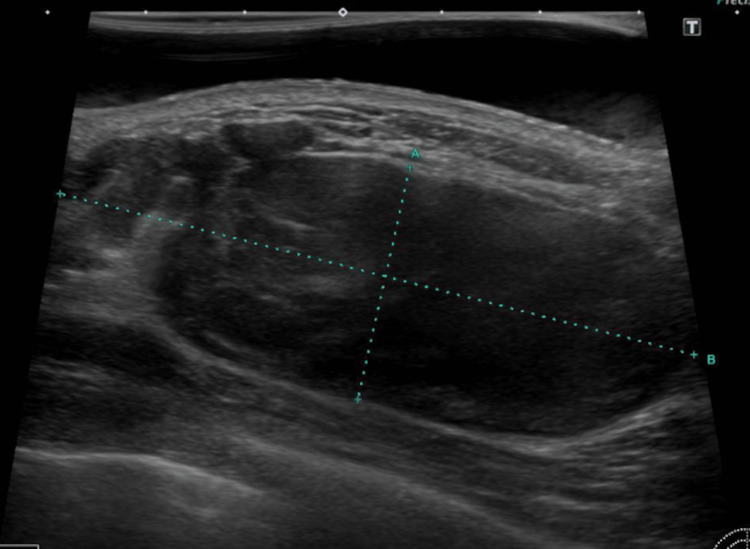
Breast ultrasonography A well-defined hypoechoic tumor sized 24 x 66 x 62 mm was observed in the left upper area

**Figure 2:**
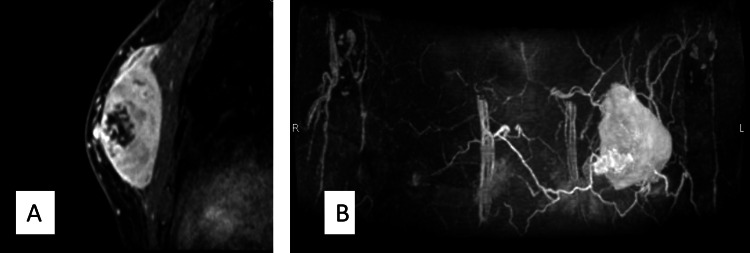
MRI of the patient (A) A 68 x 34 x 84-mm large-sized tumor with low signal intensity in fat suppression T1-weighted images (B) High signal intensity in T2- and diffusion-weighted images, and low values in an ADC map ADC: Apparent diffusion coefficient

The patient was a male in his late fifties, who consulted our medical center upon referral from a local doctor after findings of a left breast mass. A tumor sized 10 cm in diameter was observed in the upper region of the left breast. Ultrasound examination results indicated a well-demarcated hypoechoic tumor in the left upper area measuring 24 x 66 x 62 mm (Figure 1), while MRI showed a 68 x 34 x 84 mm tumor in the left chest wall, with low signal intensity in fat suppression T1-weighted images, high signal intensity in T2-weighted images, high signal intensity in diffusion-weighted images, and low values in apparent diffusion coefficient (ADC) maps. The MRI findings also revealed that the tumor was in extensive contact with the pectoralis major muscle along with possible adhesions, though there was no obvious indication of infiltration (Figure 2). Needle biopsy results were highly suggestive of sarcoma, thus the patient was referred to our hospital for treatment.

A preoperative systemic search performed with CT scanning revealed no neoplastic lesions in places other than the breast tissue, thus it was judged as a primary breast tissue tumor. A left breast resection was performed. Postoperative pathological examination results revealed the possibility of tumor cells in the resection margins, thus a left mastectomy and reconstruction of the vastus lateralis valve were performed. A follow-up examination performed two years and two months after the operation showed no evidence of recurrence or metastasis.

**Figure 3:**
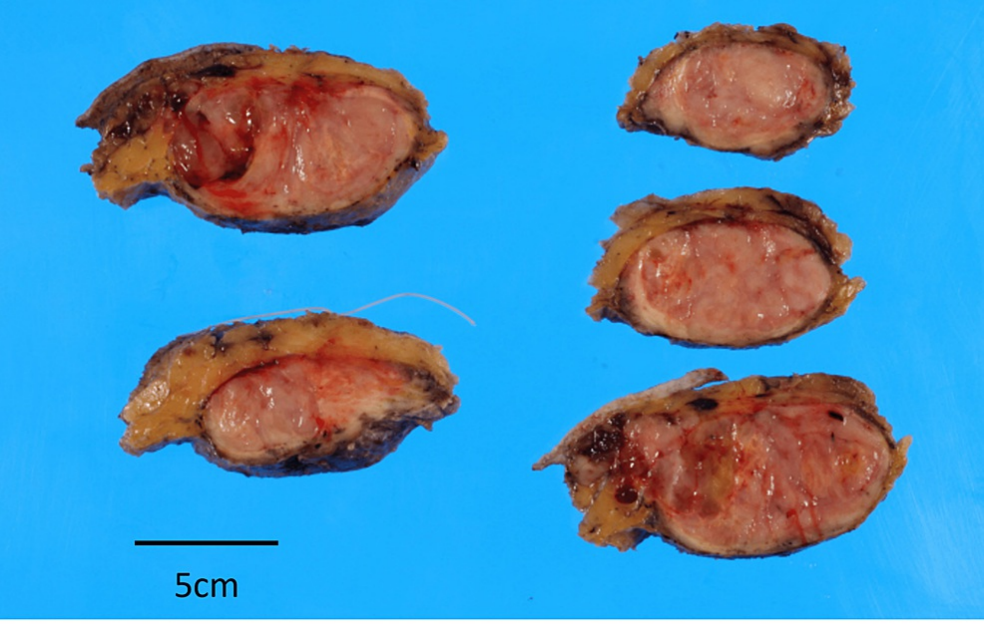
Gross findings of the resected tumor The mass was measured at 90 x 90 x 42 mm. The cut surface was heterogeneous in color and showed myxomatous areas, while the boundary of the tumor was somewhat indistinct. Infiltration into surrounding fatty and pectoralis muscle tissue was also observed.

The surgically resected tumor was measured at 90 x 90 x 42 mm. Gross findings showed the cut surface to be heterogeneous in color and mottled myxomatous areas, while the boundary was somewhat indistinct. Infiltration of tumor cells into surrounding adipose and pectoralis muscle tissues was also observed in some areas (Figure 3).

**Figure 4:**
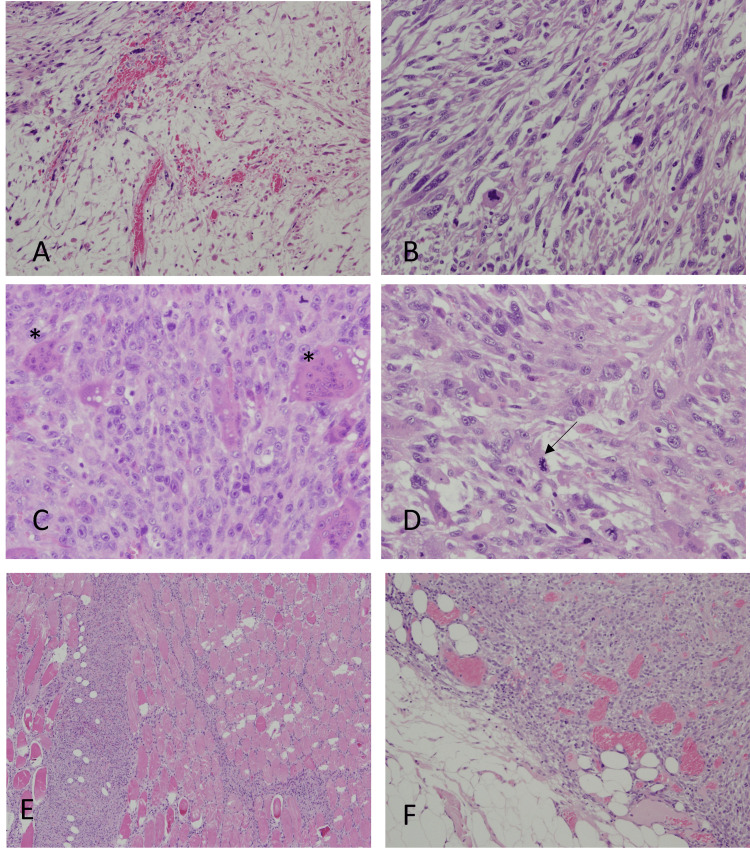
Histopathological findings (H&E staining) (A) Pleomorphic spindle-shaped cells showed proliferation in the myxoid matrix, accompanied by elongated blood vessels (B) A fibrosarcoma-like area was also observed in the tumor, including spindle-shaped tumor cells with enlarged nuclei proliferating in the myxoid matrix (C, D) Osteoclast-like giant cells (asterisks) and abnormal mitosis (arrows) were observed in the fibrosarcoma-like tumor area (E, F) Infiltration into the thoracic muscle and adipose tissue H&E: Hematoxylin and eosin

Histological findings of the tumor showed pleomorphic spindle-shaped cells proliferating in the myxoid matrix along with elongated blood vessels (Figure 4A). A fibrosarcoma-like component composed of tumor cells with enlarged nuclei was also observed, with a myxoid matrix noted between the cells (Figure 4B). Furthermore, osteoclast-like giant cells (Figure 4C) and abnormal mitosis (Figure 4D) were seen in the fibrosarcoma-like tumor area. Tumor cell infiltration into the thoracic muscle (Figure 4E) and the adipose tissue (Figure 4F) was also noted. Immunohistochemical examination results are presented in Table 1. Only vimentin was positive, while cytokeratin (CK)-AE1/AE3, S100, smooth muscle alpha-actin (α-SMA), desmin, murine double minute 2 (MDM2), cyclin-dependent kinase 4 (CDK4), cluster of differentiation (CD)117, nuclear β-catenin, B-cell lymphoma 2 (BCL-2), and CD99 were all negative. Additionally, estrogen receptor (ER), androgen receptor (AR), progesterone receptor (PgR), and CD34 were negative.

**Table 1:** Antibodies used for immunohistochemistry, and immunohistochemical results RTU: Ready to use, CK: Cytokeratin, α-SMA: Smooth muscle alpha-actin, MDM2: Murine double minute 2, IF2: Initiation factor 2, CDK4: Cyclin-dependent kinase 4, CD: Cluster of differentiation, BCL-2: B-cell lymphoma 2, ER: Estrogen receptor, AR: Androgen receptor, PgR: Progesterone receptor

Antigen	Clone	Antigen retrieval protocol	Dilution	Source	Results
CK-AE1/AE3	Cocktail of two clones, AE1/AE3	ER1 10'	RTU	Novocastra, Newcastle, UK	(-)
b-catenin	b-catenin-1	ER2 20'	1: 200	Dako, Glostrup, Denmark	(nucleus, -)
a-SMA	1A1	ER2 10'	1: 800	Dako, Glostrup, Denmark	(-)
Desmin	DE-R-11	ER2 20'	RTU	Novocastra, Newcastle, UK	(-)
Vimentin	V9	ER1 10'	1: 2400	Dako, Glostrup, Denmark	(+)
CD34	NCL-L-END	ER2 20'	RTU	Novocastra, Newcastle, UK	(-)
CD117	Polyclonal	ER2 10'	1: 400	Dako, Glostrup, Denmark	(-)
S100	Polyclonal	-	1:800	Dako, Glostrup, Denmark	(-)
ER	NCL-L-ER-6F11	ER2 20'	1:40	Novocastra, Newcastle, UK	(-)
MDM2	IF2	ER2 20'	1: 200	Life Technologies, Frederick, USA	(-)
CDK4	DCS-31	ER2 10'	1: 200	Thermo Fisher Scientific Inc, MA, USA	(-)
BCL-2	BCL-2/100/D2	ER2 20'	RTU	Leica Biosystems, UK	(-)
CD99	12E7	ER2 20'	1:400	Dako, Glostrup, Denmark	(-)
PgR	16	ER2 20'	1: 100	Novocastra, Newcastle, UK	(-)
AR	AR27	ER2 30'	1: 50	Novocastra, Newcastle, UK	(-)

## Discussion

A myxofibrosarcoma in the breast tissue is extremely rare, with only 18 known cases reported thus [1-9]. Their diagnosis has been based on histology in most cases, while various immunohistochemical markers were examined for differential diagnosis in only four (Table 2) [2,6-8]. The present report provides details of a case in which diagnosis of myxofibrosarcoma in the breast tissue was determined based on histology and immunohistochemical examination results.

Non-epithelial malignant tumors with mucinous substrate include leiomyosarcoma, synovial sarcoma, liposarcoma, chondrosarcoma, and malignant schwannoma [10-14], thus differentiation is necessary for confirming a diagnosis of myxofibrosarcoma. In all leiomyosarcomas, at least one marker of α-SMA, desmin, or h-caldesmon is positive, while two of those markers are positive in about 70% of these tumors [15].

Recently, TLE 1 was proposed as a specific marker of synovial sarcoma [16], in addition to the combination of traditional markers such as BCL-2 and CD99. Unfortunately, TLE 1 could not be performed because of a new marker that we could not obtain. Most liposarcomas show positive findings for MDM2 and CDK4 in the cell nuclei [15]. Chondrosarcomas and malignant schwannomas also have no specific markers, though S100 and CD117 have been reported positive in 20% and 30% of chondrosarcoma cases, respectively [15], while S100 and SOX10 show focal positivity in malignant schwannomas [15]. In the present case, all of the above markers were negative (Table 2). Therefore, the possibility of leiomyosarcoma, synovial sarcoma, liposarcoma, chondrosarcoma, and malignant schwannoma could be excluded based on immunohistochemical results.

**Table 2:** Cases of myxofibrosarcoma diagnosed with the help of the immunohistochemical analysis α-SMA: Smooth muscle alpha-actin, ER: Estrogen receptor, PgR: Progesterone receptor, CK: Cytokeratin, CD: Cluster of differentiation, MDM2: Murine double minute 2

No.	Immunohistochemistry	Literature
1	(+): CD34, scattered, (-): Desmin, HHF35,α-SMA, S100, ER, PgR	2
2	(+): Vimentin, (-): CK, CD45, Desmin, CD68	6
3	(-): CK (AE1/AE3)	8
4	(+): partially CD34, MDM2, (-): SMA, desmin, CDK4, CK (AE1/AE3), MUC4	7
Present case	(+): Vimentin, (-): CK-AE1/AE3, S100, αSMA, desmin, myogenin, MDM2, CDK4, p40, androgen receptor, progesterone receptor, CD34	

A malignant phyllodes tumor of the breast consists of pleomorphic spindle-shaped tumor cells that often show a mucinous matrix [17,18]. Diagnosis is relatively easy to obtain when the tumor contains a non-neoplastic epithelial component. However, malignant phyllodes tumors with an epithelial component that has been eradicated by tumor cells are difficult to differentiate when attempting to obtain a diagnosis of myxofibrosarcoma. In phyllodes tumor cases, positivity for PgR and AR have been reported in 100% [19], and 98% [20] of examined stromal cells, respectively. Sawyer et al. also reported that β-catenin was positive in the nuclei of stromal tumor cells in 72% of phyllodes tumors [21]. In the present tumor, PgR, AR, and nuclear β-catenin were all negative. Therefore, the possibility of a malignant phyllodes tumor, in this case, could be excluded.

Besides a malignant phyllodes tumor, some breast tumors with components of spindle cells with myxoid matrix or spindle cells with giant cells have to be listed for the differential diagnosis. These include myxofibroblastoma, and metaplastic carcinoma [22-24]. Especially, the exclusion of metaplastic carcinoma is very important because of the difference in the treatment strategy in advanced cases [25]. However, a component of spindle cells in metaplastic carcinoma is immunoreactive for cytokeratins though variably [22,23], while the present tumor did not express cytokeratins at all. On the other hand, myofibroblastoma is a benign tumor and its tumor cells typically express CD34, ER, PgR, and AR [15,23,24], which were not expressed in the present tumor. Therefore, both myxofibroblastoma and metaplastic carcinoma could be excluded from the differential diagnosis of the present tumor.

The five-year survival rate for a patient with myxofibrosarcoma has been reported to range from 30% to 35% [1]. Myxofibrosarcoma can metastasize to the lung, bone, and lymph node, and local recurrence following tumor resection often occurs because of an inadequate surgical procedure. Therefore, control of local recurrence by extensive tumor resection and additional radiation therapy after the resection is important [21]. Extensive tumor resection was performed in the present case, with no local occurrence or metastasis at two years and two months later.

Hartel et al. presented a clinicopathologic study of 19 cases with a primary malignant fibrous histiocytoma of the breast tissue, of which six were myxofibrosarcomas [1]. They found that such tumors were more common in middle-aged women or elderly men, with a poor prognosis for elderly patients with distant metastasis [1], as also noted in other reports of primary myxofibrosarcoma of the breast tissue [2,3,5-9]. Based on reported findings of local recurrence up to three years after surgery in similar affected cases [4], long-term follow-up examinations are considered necessary for the present patient.

## Conclusions

We presented our experience with a very rare case of primary myxofibrosarcoma of the breast. Since there are no specific immunohistochemical markers for myxofibrosarcoma, it is important to exclude non-epithelial malignancies such as leiomyosarcoma, synovial sarcoma, liposarcoma, chondrosarcoma, malignant schwannoma, myxofibroblastoma, and epithelial malignancies such as malignant phyllodes tumor, and metaplastic carcinoma. Myxofibrosarcoma has a high risk of local recurrence and metastasis to the lung, bone, or lymph node, and careful follow-up is considered necessary.
